# The role of intraoperative narrow-band imaging in transoral laser microsurgery for early and moderately advanced glottic cancer^☆^

**DOI:** 10.1016/j.bjorl.2018.01.004

**Published:** 2018-03-01

**Authors:** Hanna Klimza, Joanna Jackowska, Cesare Piazza, Jacek Banaszewski, Malgorzata Wierzbicka

**Affiliations:** a Poznan University of Medical Sciences, Department of Otolaryngology, Head and Neck Surgery, Poznan, Poland; b Fondazione IRCCS, National Cancer Institute of Milan, University of Milan, Department of Otolaryngology, Head and Neck Surgery, Milan, Italy

**Keywords:** Laser CO_2_, Early, Moderately advanced glottic cancer, Narrow-band imaging, Safe margins, Frozen sections, Laser CO_2_, Inicial, Câncer glótico moderadamente avançado, Imagens de banda estreita, Margens seguras, Cortes por congelação

## Abstract

**Introduction:**

Trans-oral laser microsurgery is an established technique for the treatment of early and moderately advanced laryngeal cancer.

**Objective:**

The authors intend to test the usefulness of narrow-band imaging in the intraoperative assessment of the larynx mucosa in terms of specifying surgical margins.

**Methods:**

Forty-four consecutive T1–T2 glottic cancers treated with trans-oral laser microsurgery Type I–VI cordectomy were presented. Suspected areas (90 samples/44 patients) were biopsied under the guidance of narrow-band imaging and white light and sent for frozen section.

**Results:**

Our study revealed that 75 of 90 (83.3%) white light and narrow-band imaging-guided samples were histopathologically positive: 30 (40%) were confirmed as carcinoma in situ or invasive carcinoma and 45 (60%) as moderate to severe dysplasia. In 6 patients mucosa was suspected only in narrow-band imaging, with no suspicion under white light. Thus, in these 6 patients 18/90 (20%) samples were taken. In 5/6 patients 16/18 (88.8%) samples were positive in frozen section: in 6/18 (33.3%) carcinoma (2 patients), 10/18 (66.6%) severe dysplasia was confirmed (3 patients). In 1 patient 2/18 (11.1%) samples were negative in frozen section. Presented analysis showed, that sensitivity, specificity and accuracy of white light was 79.5%, 20% and 71.1% respectively, while narrow-band imaging was 100%, 0.0% and 85.7%, respectively.

**Conclusion:**

The intraoperative use of narrow-band imaging proved to be valuable in the visualization of suspect areas of the mucosa. Narrow-band imaging confirms the suspicions undertaken in white light and importantly, it showed microlesions beyond the scope of white light.

## Resumo

**Introdução:**

A microcirurgia transoral a laser é uma técnica bem estabelecida para o tratamento de câncer de laringe inicial e moderadamente avançado.

**Objetivo:**

Verificar a utilidade da imagem de banda estreita na avaliação intraoperatória da mucosa laríngea na especificação das margens cirúrgicas.

**Método:**

Foram avaliados 44 cânceres glóticos T1-T2 consecutivos, tratados com cordectomia Tipo I-VI, por microcirurgia transoral a laser. As áreas suspeitas (90 amostras/44 pacientes) foram submetidas a biopsia e avaliadas através de imagens de banda estreita e luz branca e enviadas para cortes por congelação.

**Resultados:**

Nosso estudo revelou que 75 (83,3%) das 90 amostras, apresentaram histopatologia positiva na análise com luz branca e imagens de banda estreita: 30 (40%) foram confirmadas como carcinoma *in situ* ou carcinoma invasivo e 45 (60%) como displasia moderada a grave. Em seis pacientes, a mucosa apresentou-se suspeita apenas na imagem de banda estreita, sem nenhuma suspeita sob luz branca. Assim, nesses seis pacientes 18/90 (20%) amostras foram colhidas. Em 5/6 pacientes, 16/18 (88,8%) amostras mostraram resultado positivo na análise de congelação: em 6/18 (33,3%) amostras foi confirmado carcinoma (dois pacientes) e em 10/18 (66,6%) foi confirmada displasia grave (três pacientes). Em um paciente, 2/18 (11,1%) as amostras mostraram resultado negativo na congelação. A análise apresentada mostrou que a sensibilidade, a especificidade e a acurácia da luz branca foram de 79,5%, 20% e 71,1%, respectivamente, enquanto a imagem de banda estreita apresentou como resultados 100%, 0,0% e 85,7%, respectivamente.

**Conclusão:**

O uso intraoperatório de imagem de banda estreita provou ser valioso na identificação de áreas suspeitas da mucosa, confirmou as suspeitas verificadas na análise com luz branca e, o que é mais importante, identificou microlesões além do alcance da luz branca.

## Introduction

Trans-oral laser microsurgery (TLM) is an established treatment method for early-stage laryngeal tumours. On the one hand, TLM is minimally invasive and precise; on the other hand, it requires surgical experience and close cooperation with the pathologist. The most important outcome during TLM should be complete tumour resection within adequate negative margins. It has been reported that the definitions of clear margins are heterogeneous and range from 0.5 mm to 2 mm.^1^, ^2^, ^3^ Narrow band imaging (NBI) is an optical technique that enhances ability of endoscopes in evaluating the biological behaviour of a given tissue by illuminating the intraepithelial papillary capillary loops (IPCL) within its covering mucosa. The effectiveness of NBI in the early detection of squamous cell carcinoma (SCC) of the larynx has been documented.^4^, ^5^, ^6^ NBI provides superior detection of irregular microvasculature on the mucosal surface compared with conventional laryngoscopy, by better visualization of the demarcation line.^7^ Thus, superficial, dispersed or multifocal mucosal lesions that are usually not detected in conventional white-light (WL) imaging endoscopy can be identified on the basis of their vascular pattern using the NBI.^8^ According to the literature,^4^, ^9^ any well-demarcated brownish area with thick dark spots and/or winding vessels is considered a positive lesion in NBI. Furthermore, the presence of one or more afferent hypertrophic vessels branching out into small vascular loops within the lesion is considered indicative of suspect areas.

To our knowledge, there is only one paper dealing with intraoperative NBI use, exclusively concerning early glottic cancer. Garofolo et al.^10^ proved that pre- and intraoperative evaluation by high definition NBI is a very useful diagnostic tool in optimizing the evaluation of the neoplastic boundaries and in reducing the incidence of positive superficial margins after TLM. There is lack of any other proof in the available literature on benefits of intraoperative NBI use in patients with glottis cancer. Thus, we decided to design a similar study, but with wider inclusion criteria, as we enrolled in the studied group patients with confirmed T2 stage glottic cancer.

The goal of this paper is to assess the usefulness of NBI in the intraoperative imaging of the laryngeal mucosa in terms of specifying surgical margins by delineation of suspect regions beyond the scope of white light (WL) and to correlate these findings with those of histological examination.

## Methods

Forty-four consecutive patients underwent cordectomies between April 2012 and November 2013 at Poznan University of Medical Science, Tertiary Referral Head Neck Centre. To be included in the study sample, patients were required to have a TNM stage evaluation according to the 2011 American Joint Committee on Cancer International Union^11^ and histological assessment according to the WHO classification (2005). Age, sex, staging of the tumour, type of cordectomy, intraoperative frozen section (FS), and routine histology results were analysed. All patients fulfilled the inclusion criteria (squamous cell carcinoma confirmed by pathologist before cordectomy and T1–T2 stage of glottic cancer). The exclusion criterion in our study protocol was any previous treatment of glottic cancer. There was no special recruitment for the research as well as for the follow-up. All the patients were enrolled into the study during their standard treatment.

All procedures performed were in accordance with the ethical standards of the institutional research committee – Bioethics Committee of the Poznan University of Medical Science – and with the 1964 Helsinki declaration and its later amendments or comparable ethical standards. The research did not include clinical trials. All patients provided written informed consent prior to the surgery. The Bioethics Committee of the Poznan University of Medical Science approved the project of this research. Ethics committee approval no. 472/15.

### Preoperative work-up

In 5 women and 39 men, aged 54–83 years, superficial mucosa specimens were collected preoperatively and SCC was histologically confirmed. Rigid endoscopy, flexible video-endoscopy (video processor with integrated LED light source, model CV-170 with HD, ENF-VH, Olympus Corp, Tokyo, Japan) with (WL), NBI and a preoperative computer tomography scans were used to assess the larynx. Patients were classified as follows: 14/44 (31.8%) had T1a, 3/44 (6.8%) T1b, 27/44 (61.4%) had T2 cancer; all were staged N0 and M0.

### Surgical technique

All patients underwent cordectomies (Types I–VI) according to the European Laryngological Society classification with TLM under the general anaesthesia. After the endotracheal intubation, all anatomical sites were endoscopically evaluated by WL and NBI using rigid 0 and 30 angled telescopes. The light source was fitted with the optical filter for NBI to better delineate the lesion. Attention was paid to the superficial extension of the primary lesion with special emphasis to additional suspected areas in the glottis and entire larynx. In this first part of each procedure WL with magnification and NBI served to select suspicious areas, consecutively taken and sent for frozen section (FS).

The procedure was adopted in all 44 patients, and 90 samples for FS were taken. Next, the laser cordectomy was performed. Finally, the surgical specimens with margins marked in ink were sent for definitive pathology. Margins were classified as negative when greater than 0.5 mm and as positive when less than 0.5 mm.

Patients with negative margins were followed up every two months in the first year after cordectomy and every four months in the second year using videolaryngostroboscopy and trans-nasal flexible videoendoscopy with WL and NBI. Patients diagnosed with T2 lesions underwent CT examination every six months.

The main predictor variable was the number of samples taken under WL and NBI. The variables: age, sex, T category and type of cordectomy were additional predictor variables. The primary outcome variable was the comparison of histology (severe dysplasia and moderate dysplasia versus carcinoma in situ and invasive carcinoma) in FS assessment of samples guided by NBI.

Statistical analysis was performed using the Chi-squared test with the Yates correction if required, and the Student's *t*-test and Kruskal–Wallis ANOVA at a significance level of alpha = 0.05. Diagnostic test evaluation was conducted using calculation of sensitivity, specificity and accuracy.

## Results

The types of cordectomy and surgery details are presented in Table 1. Additional mucosal margins for FS were taken in all patients. Out of 90 samples, taken under the guidance of WL and NBI, 75/90 (83.3%) were histopathologically positive: 30 (40%) were confirmed as carcinoma and 45 (60%) as moderate to severe dysplasia.

**Table 1:** Summary of study characteristics – types of cordectomy and surgery details.

ID	Exact. localization of cancer	T-stage	NBI	WL	Localization of the additional samples	Extended resection	Type of cordectomy	H-P of NBI based samples	Upstage of T-stage	Follow up 24 months
1	Vocal fold right	T1a	+	+	Anterior commissure	+	Va	±	−	Free
			+	+	Subglottis			±		
2	Vocal fold left	T1a	+	+	Anterior commissure	+	Va	±	−	Free
3	Vocal fold left	T2	+	+	Anterior commissure	+	Va	±	−	Free
			+	+	Vocal fold right			±		
4	Vocal fold left	T1b	+	+	Anterior commissure	+	Va	±	+	Free
			+	+	Vocal fold right part sup.			±		
			+	+	Vestibular fold left, ventriculus laryngis left			−		
5	Anterior commissure	T2	+	+	Vocal fold right	+	Va	+	−	Free
			+	+	Vestibular fold, ventriculus laryngis right			−		
6	Vocal fold right	T2	+	+	Anterior commissure	+	VI	+	−	Free
			+	+	Vocal fold left part sup.			±		
7	Vocal fold right	T2	+	−	Anterior commissure	+	VI	+	+	Free
			+	−	Vocal fold left part sup.			+		
			+	−	Vestibular fold, ventriculus laryngis right			+		
8	Vocal fold left	T1b	+	+	Anterior commissure	−	Va	+	−	Free
			+	+	Vocal fold right in part sup.			±		
			+	+	Subglottis			−		
9	Vocal fold left	T1a	+	+	Vocal fold left part post.	−	II	±	−	Free
			+	+	Vestibular fold, ventriculus laryngis left			±		
10	Vocal fold right	T1a	+	+	Vestibular fold, ventriculus laryngis right	−	II	−	−	Free
11	Vocal fold right	T1a	+	+	Anterior commissure	−	II	−	−	Free
12	Vocal fold left	T1a	+	+	Anterior commissure	−	II	−	−	Free
13	Vocal fold left	T2	+	+	Anterior commissure	+	VI	+	+	Free
			+	+	Vocal fold right part sup.			+		
14	Vocal fold right	T1a	+	+	Anterior commissure	−	III	±	−	Free
15	Vocal fold right	T1a	+	+	Anterior commissure	−	II	±	−	Free
16	Anterior commissure	T2	+	+	Others	+	Vc	+	+	Free
17	Vocal fold right	T2	+	+	Anterior commissure	−	Va	±	−	Free
			+	+	Vestibular fold, ventriculus laryngis right			±		
18	Vocal fold right	T2	+	+	Anterior commissure	+	Va	+	−	Free
19	Vocal fold left	T2	+	+	Anterior commissure	−	Va	±	−	Recurrence
			+	+	Vocal fold right part sup.			±		
20	Vocal fold right	T2	+	+	Anterior commissure	+	Va	±	−	Free
			+	+	subglottis			±		
21	Vestibular fold right	T2	+	+	Anterior commissure	+	VI	±	+	Free
			+	+	Vocal fold left part sup.			±		
22	Vocal fold left	T2	+	+	Anterior commissure	+	VI	+	+	Free
			+	+	Vocal fold right part sup.			+		
23	Vocal fold right	T2	+	+	Anterior commissure	+	Va	+	+	Free
			+	+	Vocal fold left part sup.			±		
24	Vocal fold right	T2	+	+	Anterior commissure	+	Va	±	+	Free
			+	+	Vestibular fold, ventriculus laryngis right			+		
25	Vocal fold right	T1a	+	+	Subglottis	−	III	±	−	Free
26	Vocal fold left	T2	+	+	Anterior commissure	+	Vb	±	−	Free
			+	+	Others			+		
27	Vocal fold left	T1a	+	−	Anterior commissure	+	IV	±	−	Free
			+	−	Vocal fold right			±		
			+	−	Vestibular fold, ventriculus laryngis left			±		
28	Vocal fold left	T1a	+	+	Anterior commissure	−	I	−	−	Free
			+	+	Vocal fold left part post.			±		
29	Vocal fold left	T1a	+	+	Vocal fold left part post.	−	I	±	−	Free
			+	+	Vocal fold right part post.			±		
30	Vocal fold right	T2	+	−	Anterior commissure	−	IV	−	−	Free
			+	−	Subglottis			−		
			+	−	Vestibular fold, ventriculus laryngis right			−		
31	Vocal fold left	T2	+	−	Anterior commissure	−	VI	±	−	Free
			+	−	Vocal fold right part sup.			±		
			+	−	Vestibular fold, ventriculus laryngis left			±		
32	Vocal fold left	T1a	+	+	Vocal foldleft part sup.	−	III	+	−	Free
			+	+	Subglottis			±		
33	Vocal fold right	T2	+	+	Anterior commissure	+	Va	±	+	Free
			+	+	Vocal fold right part sup.			±		
			+	+	Vestibular fold, ventriculus laryngis right			+		
34	Vestibular fold right	T2	+	+	Vocal fold right	+	Vb	±	−	Free
			+	+	Vocal fold left part sup.			−		
			+	+	Others			±		
35	Vocal fold right	T2	+	+	Anterior commissure	−	VI	±	−	Free
			+	+	vestibular fold, ventriculus laryngis right			±		
36	Vocal fold right	T1a	+	+	Anterior commissure	−	IV	−	−	Free
37	Vocal fold left	T1b	+	+	Anterior commissure	+	Va	+	−	Free
			+	+	subglottis			−		
38	Vocal fold right	T2	+	−	Anterior commissure	+	VI	+	+	Free
			+	−	Vocal fold left part sup.			+		
			+	−	Subglottis			±		
39	Vocal fold left	T2	+	+	Anterior commissure	+	IV	+	−	Free
			+	+	Vestibular fold, ventriculus laryngis left			+		
40	Anterior commissure	T2	+	−	Vocal fold right part middle	+	VI	+	+	Free
			+	−	Vocal fold left part middle			±		
			+	−	Vestibular fold, ventriculus laryngis right			±		
41	Vocal fold left	T2	+	+	Anterior commissure	+	Vb	−	−	Free
			+	+	Others			+		
42	Anterior commissure	T2	+	+	Subglottis	+	Vd	±	−	Free
			+	+	Vestibular fold, ventriculus laryngis left			+		
			+	+	Others			+		
43	Vocal fold left	T2	+	+	Anterior commissure	+	VI	+	+	Recurrence
			+	+	Vocal fold right part sup.			+		
			+	+	Vestibular fold, ventriculus laryngis left			−		
44	Vocal fold right	T2	+	+	Anterior commissure	+	VI	+	+	Recurrence
			+	+	Vocal fold left part sup.			+		

±, neoplasia; −, negative; +, cancer.

Extended resection – extension of superficial margins if an intraoperative histology was ± or +.

**Figure 1:**
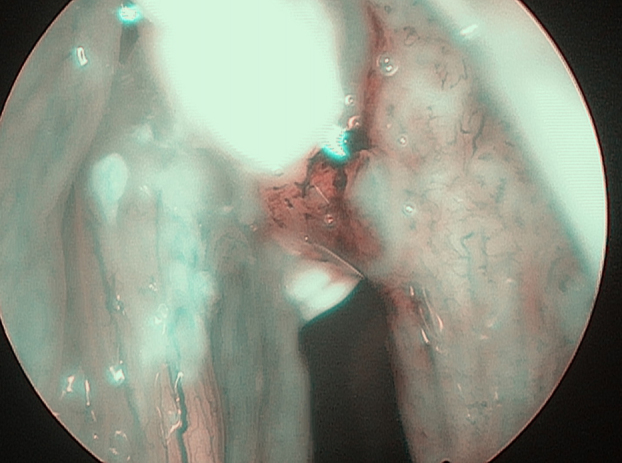
Glottic cancer in narrow-band imaging (NBI).

**Figure 2:**
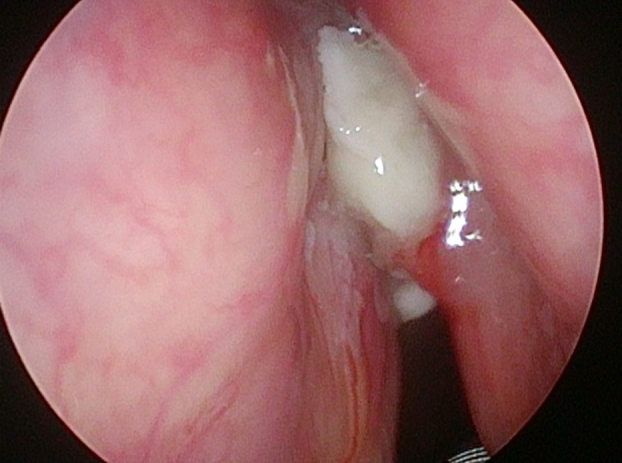
Glottic cancer in white light (WL).

In 6 patients mucosa was suspected only in NBI (Fig. 1), with no such evidence in WL (Fig. 2). In these 6 patients 18/90 (20%) samples were taken. In 5/6 patients 16/18 (88.8%) samples were positive in FS. In 6/18 (33.3%) samples (2 patients) carcinoma, in 10/18 (66.6%) samples (3 patients) severe dysplasia were confirmed. One patient in 2/18 (11.1%) samples had a negative FS.

To summarize, in 6/44 (13.6%) patients NBI allowed to take the FS, so that the diseased mucosa was included into the surgical specimens. Presented analysis showed that sensitivity, specificity and accuracy of WL was 79.5%, 20%, and 71.1% respectively, while NBI corresponding values were: 100%, 0.0%, and 85.7%, respectively.

There was no interrelation between T-category and number of NBI guided samples (Chi2 [4] = 5.17, 0.26982). In the anterior part of the vocal fold the rate of additional anterior commissure sampling was significantly higher (Chi2 [4] = 10.22, 0.03689) than in other anatomical sites of the glottis, but there were no differences in the ratio of positive/negative FS.

In final pathology, negative margins were obtained in all patients. Local recurrence was found in 3/44 (6.8%) patients after 18, 26, and 30 months of follow up, respectively. Two out of them underwent recordectomy, whereas one underwent a partial laryngectomy. Open partial laryngectomy was required in 2 patients, whereas one patient required a second TLM. Forty-one out of forty-four (93.1%) patients were recurrence free at the last follow up (ranging from 18 to 36 months). There was no interrelation between the recurrence and: age (T [41] = 1.57, *p* = 0.124565), gender (Chi2 [1] = 0.51, *p* = 0.47590), T-category (Chi2 [1] = 2.19, *p* = 0.4182), primary localization of the tumour (Chi2 [1] = 0.17, *p* = 0.6838), number of additional samples in NBI (Chi2 [4] = 1.80, *p* = 0.77298), and the histology of NBI guided samples (Chi2 [2] = 2.33, *p* = 0.31146).

## Discussion

TLM is the predominant method for management of early laryngeal cancer^12^ and is also advocated for more advanced lesions^10^, ^13^, ^14^, ^15^ but the impact of margin status on the outcome is still controversial.^16^, ^17^, ^18^ Peretti et al.^1^ and Jackel et al.^19^ confirmed the influence of positive margins on local recurrence. In contrast, Hoffmann et al.^18^ found that “second look” laryngoscopy showed a low rate of residual cancer, even in margin-positive patients.

The majority of authors underscore that safe margins are indispensable to obtaining a successful outcome of TLM,^15^ but the problem of how to better mark or improve the demarcation of safe margins remains unsolved. One drawback of TLM is the difficulty in the interpretation of histological specimens as a consequence of the thermal effect of lasers on tissues. Laser margin excision is associated with a greater degree of artefact than cold steel instrument excision, although it is not associated with a lower rate of interpretability.^20^ One of the crucial factors that influences free margin endings after cordectomy is close cooperation with pathologists. The purpose of this study, which was to assess the histology of margins guided by NBI and WL examination and the influence of NBI on intraoperative decision making, was fulfilled and showed the superiority of NBI over WL.

Even intraoperatively, it is still difficult to visualize cancer foci separated from the main lesion by WL endoscopy alone, because most of them are isochromatic or show a flat shape. Mucosal and submucosal vessel networks on NBI view is in striking contrast to the surrounding mucosa, which is a distinguishing feature of NBI view and is rarely obtained in conventional WL. Under NBI, the image of submucosal vessels near the edging area may be abnormal, which may lead to a wider excision. NBI could recognize demarcated abnormal intraepithelial microvascular changes within laryngeal lesions, which were hardly recognizable on conventional WL. Orita et al.^21^ presented a case of superficial hypopharyngeal cancer whose surgical extent was estimated by NBI. The activity of NBI seems to be helpful also in dealing with the resection margin during oropharyngeal and hypopharyngeal surgery.^21^

To the best of our knowledge, there is only one paper dealing with intraoperative NBI assessment, exclusively concerning early glottic cancer. According to Garofolo et al.^10^ pre- and intraoperative evaluations by high definition NBI are very useful diagnostic tools to optimize the evaluation of the neoplastic boundaries and to reduce the incidence of positive superficial margins after TLM. However, they highlight a very important role of the learning curve in this technique. They found increased accuracy of neoplastic superficial spreading evaluation during TLM in 82 Tis–T1a glottic cancer patients. Our paper includes and compares T1 and T2 patients, and NBI proved to be even more helpful in more advanced tumours.

In our study, we confirmed a very high sensitivity of NBI (100%). Our results are comparable with other authors. In studies performed by Piazza et al.^4^ and Zabrodsky et al.^22^ this value reached 100% as well. The superiority of NBI over WL is proved by low sensitivity of WL alone endoscopy in our group (79.5%). In the study carried out by Piazza et al.^4^ sensitivity of WL reached comparable value (66%). As one of our inclusion criteria was squamous cell carcinoma confirmed by pathologist before cordectomy, there were no results we could classify as false positive. Thus, specificity values in our group are very low.

Our study has some limitations. The main endpoint in fact was to compare the diagnostic advantage of NBI in the assessment of intraoperative margins, thus confirming the overall accuracy of these techniques already observed by others.^4^, ^10^ Therefore, we did not enrol patients into a control group, because all glottic cancers in last two years were given NBI intraoperative diagnostics. Although our series comprised 44 patients, the sample was not homogeneous. The unique feature of our study is the inclusion of the whole spectrum of early and moderately advanced glottic tumours (T1–T2b) and different types of cordectomies (I–VI).

On the basis of the analysis of our patients, we concluded that NBI can be recommended to improve intraoperative decision-making concerning delineation of the superficial resection margins in TLM procedures for early and moderately advanced glottic cancers. The use of NBI can potentially limit the need for “second look” surgeries.

## Conclusion

Routine use of intraoperative narrow band imaging increases the accuracy of safe margins in T1 and T2 glottic cancer treated with trans-oral laser microsurgery.

## Compliance with ethical standards

All procedures performed in the study were in accordance with the ethical standards of Poznan University of Medical Science and with the 1964 Helsinki declaration and its later amendments or comparable ethical standards. The study was approved by Bioethical Committee of Poznan University of Medical Science.

## Conflicts of interest

The authors declare no conflicts of interest.
